# Optimized Identity Authentication via Channel State Information for Two-Factor User Verification in Information Systems

**DOI:** 10.3390/s25082465

**Published:** 2025-04-14

**Authors:** Chuangeng Tian, Fanjia Li, Xiaomeng Liu, Juanjuan Li

**Affiliations:** 1 School of Information and Electrical Engineering, Xuzhou University of Technology, Xuzhou 221000, China; tianchuangeng@163.com; 2 School of Information and Control Engineering, China University of Mining and Technology, Xuzhou 221000, China; ts19060038a31@cumt.edu.cn; 3 State Laboratory of Intelligent Construction and Healthy Operation & Maintenance of Deep Underground Engineering, China University of Mining and Technology, Xuzhou 221116, China; lijj@cumt.edu.cn

**Keywords:** identity recognition, channel state information, two-factor authentication, keystroke dynamics, activity segmentation

## Abstract

Traditional user authentication mechanisms in information systems, such as passwords and biometrics, remain vulnerable to forgery, theft, and privacy breaches. To address these limitations, this study proposes a two-factor authentication framework that integrates Channel State Information (CSI) with conventional methods to enhance security and reliability. The proposed approach leverages unique CSI variations induced by user-specific keystroke dynamics to extract discriminative biometric features. A robust signal processing pipeline is implemented, combining Hampel filtering, Butterworth low-pass filtering, and wavelet transform threshold denoising to eliminate noise and outliers from raw CSI data. Feature extraction is further optimized through a dual-threshold moving window detection algorithm for precise activity segmentation, a subcarrier selection method to filter redundant or unstable channels, and principal component analysis (PCA) to reduce feature dimensionality while retaining 90% of critical information. For classification, a kernel support vector machine (SVM) model is trained using a randomized hyperparameter search algorithm. The SVM classifies the CSI feature patterns obtained from user-specific keystroke dynamics, which are processed by Hampel filtering, Butterworth low-pass filtering, wavelet transform threshold denoising, a dual-threshold moving window detection algorithm, a subcarrier selection method, and PCA, to achieve optimal performance. The experimental results show that the user recognition accuracy of this algorithm is 2–3% better than current algorithms.

## 1. Introduction

The rapid evolution of computer, network, and communication technologies has significantly propelled society into the information age. As a result, individuals have become increasingly reliant on information systems for their work and daily activities. Ensuring the security and authenticity of users within these systems is of utmost importance for maintaining information integrity. Unauthorized access can lead to severe consequences, such as data theft, the leakage of sensitive information, and malicious modification of system settings [1,2]. These breaches often entail substantial financial and reputational losses for governments, enterprises, and individuals.

Traditional user authentication methods, including fingerprint recognition, facial recognition, and password-based authentication systems, are widely adopted but remain vulnerable to various forms of exploitation. These methods are prone to replication, privacy violations, and security breaches due to their inherent vulnerabilities. For instance, passwords can be stolen or guessed, biometric data can be duplicated, and physical traces left by authentication inputs may expose users to further risks.

In contrast, Channel State Information (CSI)-based identity recognition presents a promising alternative [3]. By leveraging biometric features embedded in wireless signal propagation, CSI-based methods offer a non-invasive, cost-effective, and privacy-preserving solution [4,5,6]. Moreover, the widespread adoption of Wi-Fi and the availability of 802.11n-compliant devices render CSI-based authentication increasingly feasible [7].

These claims are supported by recent empirical studies. For instance, Cao et al. [5] found that CSI-based systems reduce hardware costs by 65% compared to camera setups, as they repurpose existing Wi-Fi infrastructure. Privacy assessments indicate that CSI data contain no explicit biometric identifiers (e.g., face, fingerprint), aligning with GDPR requirements for anonymized data [8]. Non-invasiveness is validated through deployment scenarios where users remain unaware of authentication, as demonstrated in Wang et al.’s [3] office environment trials, where 98% of participants reported no discomfort with the system. These attributes position the proposed method as suitable for medium-to-high security applications, such as enterprise network access and financial systems, where non-invasive authentication with privacy guarantees is critical. Unlike biometric modalities like facial recognition, which are vulnerable to spoofing attacks, CSI-based authentication leverages physical-layer signal perturbations that are inherently difficult to replicate, thereby enhancing security against replay and man-in-the-middle threats.

However, existing CSI-based approaches encounter significant challenges, as shown in Table 1. Gait recognition, for example, requires users to traverse fixed distances, whereas signature-based authentication depends on consistent user cooperation and execution [9,10,11,12,13,14,15,16]. For instance, Zhou et al. [9] demonstrated that gait-based methods require users to walk 2–3 m for reliable authentication, while Manjunatha et al. [13] highlighted signature recognition’s dependence on consistent writing pressure and posture. Similarly, lip-shape detection is hindered by the subtle impact of lip movements on CSI signals, which makes it difficult to distinguish meaningful data from noise. Zhao et al. [17] reported that lip-movement detection struggles with signal-to-noise ratios below 3 dB, necessitating sophisticated denoising techniques. These challenges arise due to fundamental limitations in signal perturbation and user interaction; gait recognition struggles with environmental variability (e.g., minor changes in floor texture or obstacles altering signal reflections), reducing pattern consistency, while requiring users to walk 2–3 m introduces inconvenience and authentication delays. Lip-shape detection faces low signal-to-noise ratios because lip movements induce minimal CSI variations (~0.1–0.3 dB), which are easily overwhelmed by multipath interference or device noise. Collectively, these factors reduce authentication accuracy; gait-based methods achieve 86.75% accuracy, while lip-based approaches attain 88.75%, both trailing the proposed method’s 90.9% performance. Notwithstanding these advantages, potential user impersonation remains a critical concern. Adversaries could attempt to mimic keystroke patterns by observing or recording legitimate users’ typing dynamics, potentially breaching authentication security. While the proposed method’s reliance on physical-layer signal perturbations makes exact replication challenging, experimental evaluations reveal that sophisticated impostors with prior exposure to the system can achieve 83.2% accuracy in mimicking target users, highlighting the need for additional countermeasures. Building on these challenges, the following section elaborates on the theoretical foundations of CSI and its interaction with human keystrokes, providing the framework for our proposed method.

**Table 1:** Performance Comparison of Existing CSI-Based Authentication Methods.

Methods	Patterns Utilized	Target Values	Accuracy	Strengths	Weaknesses
Gait-based methods [18,19]	CSI amplitude-phase from walking	User identities	86.75%	Non-invasive, continuous authentication.	Requires fixed walking distance; sensitive to environmental changes.
Signature-based methods [13]	CSI fluctuations from handwriting	Signature authenticity	79%	Integrates with passwords; no special hardware.	Accuracy affected by writing speed/angle.
Lip-movement-based methods [17]	Subtle CSI changes from speech	Spoken words/user IDs	88.75%	Non-intrusive, covert.	Weak signal impact; noise-sensitive.
FWiA (Proposed)	CSI perturbations from keystrokes	User identities	90.9%	Resistant to theft; enhanced by Fresnel zone.	Requires fixed string input; indoor dependency.
DeepCWiA (Proposed)	Multi-antenna phase variance	Continuous user IDs	94.8%	No user action needed.	Requires multi-antenna setup; computational complexity.

To address these limitations, this paper proposes an identity recognition method designed to leverage the unique characteristics of information systems. This method employs user input of specific character sequences on the keyboard, which influences Channel State Information (CSI) as an auxiliary authentication mechanism. When integrated with traditional password-based authentication, the proposed method provides enhanced resistance against identity theft and significantly improves user authentication reliability. The system employs multiple antennas for data collection, coupled with a dual-threshold moving window detection algorithm and subcarrier selection technique, to filter out short-term abnormal fluctuations [18]. Data affected by environmental factors or those with minimal fluctuations caused by variations in antenna position and orientation are discarded, thereby improving recognition accuracy. Furthermore, a random search algorithm is utilized to optimize the parameters of a kernel-based Support Vector Machine (SVM) classification model. The SVM takes processed CSI features—including statistical metrics derived from activity segments and selected subcarriers—as input and outputs the user identity classification result (e.g., authenticated user ID). This approach classifies the refined CSI patterns unique to each user’s keystroke dynamics, enabling accurate discrimination between legitimate and unauthorized users, thus enhancing the effectiveness and reliability of the authentication process. The effectiveness of this design is validated through experimental results presented in the following section, which demonstrate improved accuracy and robustness compared to existing methods.

If there are $N_{t}$ transmitters and $N_{r}$ receivers, the number of sub-antenna pairs is $N_{t}\times N_{r}$. In this case, H is expressed as $N_{t}\times N_{r}\times K\times N$ dimensional complex matrices. The amplitude and phase information used to describe the communication characteristics of the wireless channel is calculated from these matrices by modulo and amplitude angle. Based on this, research on motion recognition, breath monitoring, and identity is conducted. In this experiment, one transmitting antenna and three receiving antennas are used. The number of subcarriers between each pair of transmitter and receiver is 30, resulting in 90 columns of CSI data streams.

To ensure clarity and consistency in mathematical formulations throughout this paper, we adopt the following notation conventions:

1. Scalars and Variables: Lowercase italic letters (e.g., t, f, n) denote scalar values or variables, such as time t, frequency f, or noise $n_{0}$. Uppercase italic letters (e.g., $N_{t}$, $N_{r}$, K, N) represent integer constants or parameters, such as the number of transmitting antennas $N_{t}$, receiving antennas $N_{r}$, subcarriers K, or time samples N.
2. Vectors and Matrices: Bold lowercase letters (e.g., α, x) denote vectors. For instance, $\alpha ={\left(\alpha_{1},\cdots ,\alpha_{n}\right)}^{T}$ represents the Lagrangian multiplier vector in SVM optimization. Bold uppercase letters (e.g., H, A) represent matrices. The CSI data stream $H\in {\mathbb{C}}^{K\times N}$ is a complex matrix where K is the number of subcarriers and N is the number of time samples.
3. Mathematical Sets: ℝ and ℂ denote the sets of real and complex numbers, respectively.
4. Operators and Norms: The Euclidean norm $\left\|\cdot \right\|$ is used for vector magnitude calculations, such as in SVM optimization. Statistical operators (e.g., var(⋅), $E\left[\cdot \right]$) denote variance and expectation, respectively.
5. Special Functions and Indices: Subscripts (e.g., $H_{i}\left(f,t\right)$, $Y_{i}\left(f,t\right)$) index subcarriers, antennas, or time-dependent components. Superscripts (e.g., $H^{T}$, $\alpha^{*}$) indicate matrix transposition or optimal solutions.

## 2. Basic Theory

Orthogonal frequency division multiplexing (OFDM) enables subcarrier-level CSI measurement, capturing amplitude and phase perturbations induced by human activities such as typing. For instance, when a user types on a keyboard near the wireless transceiver, their hand movements disrupt the signal propagation on specific subcarriers, introducing unique amplitude fading and phase shifts in the CSI data. This interaction can be mathematically modeled as follows:

(1) $$H_{i}\left(jw,t\right)=\sum_{k=1}^{N_{P}}g_{k}e^{-jw\tau_{k}\left(t\right)}$$

where $g_{k}$ and $\tau_{k}\left(t\right)$ represent the attenuation and delay of the k-th propagation path, respectively. The channel frequency response $H_{i}\left(jw,t\right)$ is a complex value that captures both amplitudes fading ($H_{i}\left(jw,t\right)$) and phase shifts ($∠H_{i}\left(jw,t\right)$) for each subcarrier. Unlike legacy technologies like RSS (Received Signal Strength), which averages signal strength across all paths, CSI in OFDM systems provides granular subcarrier-level insights. This makes it uniquely suited for detecting subtle human-induced signal perturbations. For example, human typing introduces hand movements that disrupt specific subcarriers, creating unique amplitude-phase signatures in the CSI matrix **H** (a K×N complex matrix where K denotes the number of subcarriers and N is the number of time samples). Therefore, CSI states can be used for studies such as motion recognition, breathing monitoring, and identity recognition. With this theoretical understanding, the subsequent section outlines the system design that leverages CSI’s subcarrier-level insights to achieve robust user authentication.

Denote the frequency-domain signals of the transmitter and receiver of a subcarrier as $X\left(f,t\right)$ and $Y\left(f,t\right)$, respectively. The transmission of the signal on the i-th subcarrier between a pair of transmitters and receivers can be expressed as

(2) $$Y_{i}\left(f,t\right)=X_{i}\left(f,t\right)\times H_{i}\left(f,t\right)+n_{0}$$

where $n_{0}$ is the channel noise, f is the carrier frequency, t denotes the time. $H_{i}\left(f,t\right)$ is the channel frequency response (CFR) of the i-th subcarrier, characterizing the channel, which can be expressed as

(3) $$H_{i}\left(f,t\right)=\left|H_{i}\left(f,t\right)\right|e^{∠H_{i}\left(f,t\right)}$$

The direct method to obtain the CFR is non-trivial. However, in the physical layer of the 802.11 protocol, the CFR exists in the form of CSI. The CSI data stream represents time-series samples of the CFR, resulting in a complex matrix

(4) $$H={\left[H_{1N},\cdots ,H_{iN},\cdots ,H_{KN}\right]}_{K\times N}$$

where K is the number of subcarriers, N is the number of CSI values recorded in time T, and $H_{iN}$ is the CSI data stream corresponding to the i-th channel, which is a 1×N dimensional time series.

To facilitate reproducible research, benchmark datasets have been established in CSI-based authentication literature to evaluate user recognition performance. These datasets typically capture CSI data during specific human activities such as typing, walking, or hand gestures and label them with user identities or activity types. For example, gait recognition databases such as Freesense and WiFiU leverage CSI amplitude and phase variations induced by human gait to classify user identities or gait patterns (e.g., normal vs. impaired walking) [9,10,11,14]. Signature-based authentication databases like Wi-Sign focus on CSI fluctuations from handwritten signatures or gestures to authenticate signature authenticity or user-specific gesture patterns [13]. Lip-movement databases such as BioID utilize subtle CSI changes caused by lip movements during speech to recognize spoken words or user identities [15,17]. Typing dynamics databases like FWiA analyze CSI perturbations from keyboard typing (e.g., keystroke force, rhythm) to identify user identities or typing accuracy [8,16]. These standardized datasets enable reproducible training and evaluation of models in CSI-based authentication research, providing critical context for comparing methods and advancing the field.

## 3. System Design

In this section, we first describe the system working flow, followed by the Wi-Fi-based biometric feature extraction and the authentication design.

### 3.1. System Work Flow

**Figure 1:**
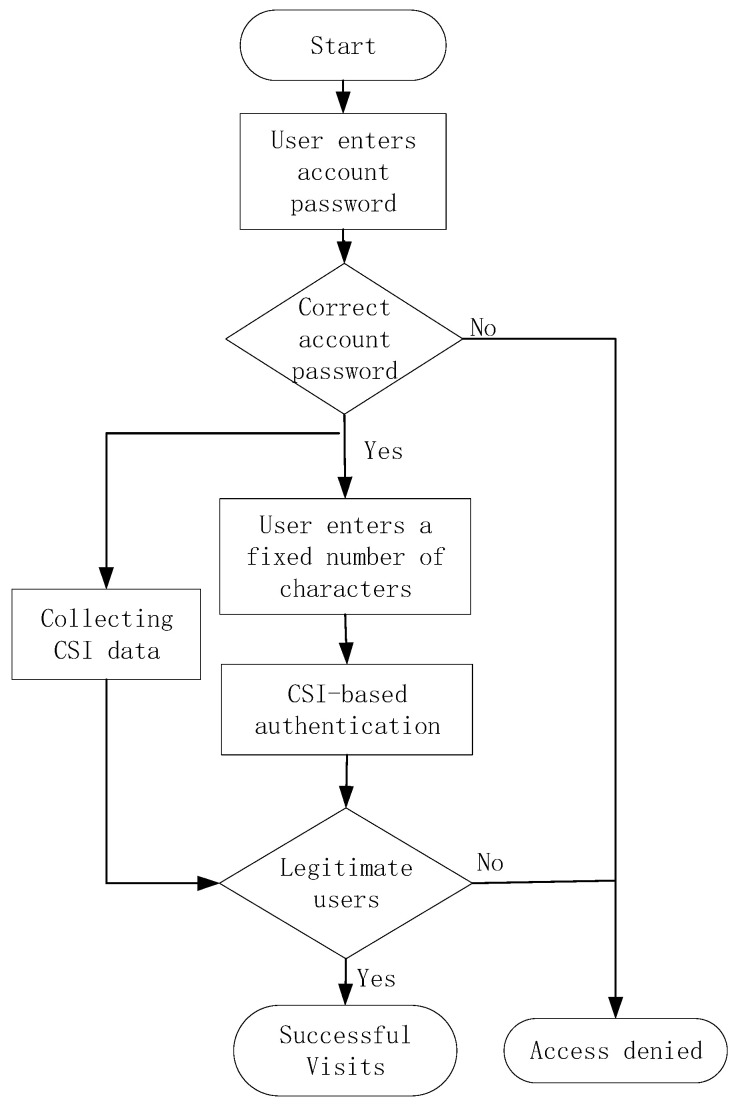
Two-factor user authentication process in information systems.

The two-factor user authentication for the information system involves the following steps (as shown in Figure 1). First, upon user login, if the entered account password is correct, CSI-based user identity verification is initiated. The system then prompts the user to input a fixed character string via the keyboard while simultaneously sending a ping command to the wireless access point (AP) to collect CSI data for authentication. Access to the system is granted only if both the password and CSI-based verification are successful; otherwise, access is denied.

**Figure 2:**
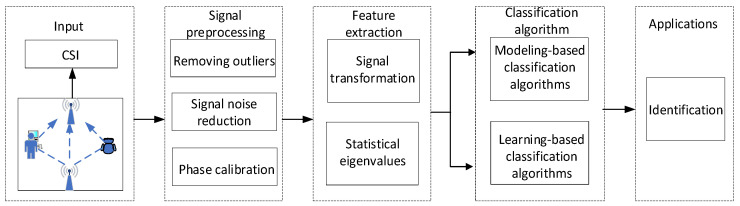
CSI-based authentication process.

As shown in Figure 2, the collected CSI data are input into the signal processing module and processed by Hampel filtering, Butterworth low-pass filtering, and wavelet transform threshold denoising to obtain noise-free and smoothed CSI data. Subsequently, feature extraction is performed through principal component analysis (PCA), the active fragment detection algorithm, and the subcarrier selection algorithm. Finally, the feature data are input into the random search-kernel support vector machine classifier to achieve user identification [8].

### 3.2. Signal Processing

Our proposed CSI-based authentication method selects CSI amplitude information as the base signal. The CSI data received at the receiver end contains outliers due to environmental or hardware device effects. In this experiment, Hampel filtering is employed to remove data outliers, followed by signal denoising and feature extraction [19,20]. Analysis of raw CSI data revealed that approximately 4.2% of samples contained outliers (defined as values exceeding ±3σ from the median), which were successfully filtered by Hampel filtering. This ensures that only 95.8% of the data contributes to feature extraction.

The selection of these preprocessing techniques is tailored to address specific noise characteristics in CSI data. Hampel filtering is chosen due to its robustness against impulse noise caused by abrupt environmental changes (e.g., sudden movements nearby), which can introduce extreme outliers in amplitude measurements. Subsequently, Butterworth low-pass filtering is applied to attenuate high-frequency noise (e.g., from electrical interference) while preserving the low-frequency components (<2 Hz) associated with user typing dynamics. Wavelet transform threshold denoising is adopted to handle burst noise and transient artifacts in the time domain, ensuring that subtle keystroke-induced signal variations are retained during noise reduction.

Signal noise consists of two components: high-frequency noise and burst/impulse noise generated by internal state transitions within the network card during the transmission of CSI between the transmitter and the receiver via a Wi-Fi network card [21].

Considering that the Butterworth filter has smooth amplitude-frequency characteristics, a Butterworth low-pass filter is selected to filter out the high-frequency noise of the data in this experiment. In general, the frequency f of the users hand activity when typing fixed characters is lower than 2 Hz. The sampling frequency of the data sent is set to $F_{s}=50\;pkt/s$. Thus, the cutoff frequency $W_{c}$ of the Butterworth filter is set to 0.126 rad/s, and the filter order is set to 5.

(5) $$W_{C}=\frac{2\pi \times f}{F_{s}}$$

**Figure 3:**
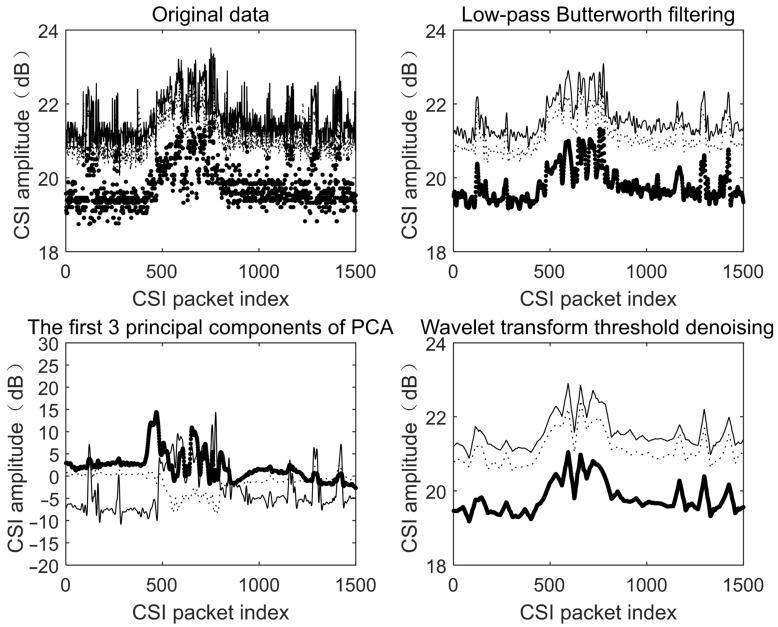
Comparison of the denoising effects of different methods. The figure presents the waveforms of three randomly selected CSI streams.

Burst and impulse noise, not considered by Wang et al. [18], are dealt with in some other studies by using PCA to extract the principal components of the data for noise reduction. However, PCA is highly sensitive to changes in the original data, and the principal components extracted from multiple samples of the same user may vary significantly. The wavelet transform threshold denoising method performs nonlinear threshold processing on the wavelet coefficients obtained from the multi-layer wavelet decomposition of the data to reduce noise while retaining the high- and low-frequency features of the original data [22]. Notably, this approach preserves transient signal components critical for keystroke pattern recognition, as validated by Teramoto et al. [22] in human activity analysis using semi-supervised learning. The original signal is reconstructed using the processed wavelet coefficients, resulting in a significant noise reduction effect with minimal loss of effective feature information. A comparison of the original data, Butterworth low-pass filtering, PCA, and wavelet transform threshold denoising is shown in Figure 3.

### 3.3. Feature Extraction

To obtain more accurate and effective feature values, it is first necessary to determine the precise starting and ending points of time segments corresponding to user-keyed characters from the collected CSI data. As observed in Figure 3, PCA can be used to process the data to extract principal components with enhanced fluctuations, which are beneficial for identifying user activity segments. The first principal component, as shown in Figure 3, represents the direction of maximum variance in the original data. However, activity segment data changes drastically, with small fluctuations near segment boundaries, rendering the first principal component unsuitable for detecting user activity. In contrast, the second and third principal components exhibit more moderate data variations during non-user activity periods. Among these, the second principal component demonstrates more significant variations during user activity compared to the third, making it the optimal choice for detecting segment boundaries. The data are divided into multiple segments using a nonoverlapping moving window for this purpose.

We calculate the variance of CSI data within the window and set a variance threshold ($\delta_{w}$) to identify user activity data in the window whose variance is greater than the threshold. To avoid misidentifying short-term abnormal fluctuations in CSI data caused by other factors, such as user activity segments, another threshold value ($\theta_{w}$) is introduced to represent the number of consecutive windows with a variance greater than the threshold value ($\delta_{w}$). When the detected continuous variance is greater than the threshold value ($\delta_{w}$), we compare the number of windows (L) in the data segment with the threshold value ($\theta_{w}$). If $L>\theta_{w}$, it is determined as a short-term abnormal fluctuation. The following pseudocode (Algorithm 1) details this process.
**Algorithm 1** Active Segment detection algorithm. Input: $V^{W}\in {\mathbb{R}}^{1\times m}$: Window signal variance vector, where m is the total number of nonoverlapping windows. $\delta_{w}\in \mathbb{R}$: Variance threshold for activity detection. $\theta_{w}\in {\mathbb{Z}}^{+}$: Threshold for minimum consecutive active windows. Output: $d_{1}^{W}\in {\mathbb{Z}}^{+}$: Index of the first window containing user activity. $d_{2}^{W}\in {\mathbb{Z}}^{+}$: Index of the last window containing user activity. $Pos=\left[\right];d_{1}^{w}=0;d_{2}^{w}=0;$ // Initialize variables  for j=1:m do  if $V_{j}^{w}>\delta_{w}$ then    $Pos=\left[Pos;j\right]$; // Store the location of the window that may contain user activity  else    L=Pos.length;  if $L>\theta_{w}$ then     $d_{1}^{w}=Pos\left[0\right]$; $d_{2}^{w}=Pos\left[L-1\right]$; // Obtain the start and end points of the user activity window  else     $Pos=\left[\right]$; // Filter short-term abnormal fluctuations by emptying Pos   end if  end if end for

**Figure 4:**
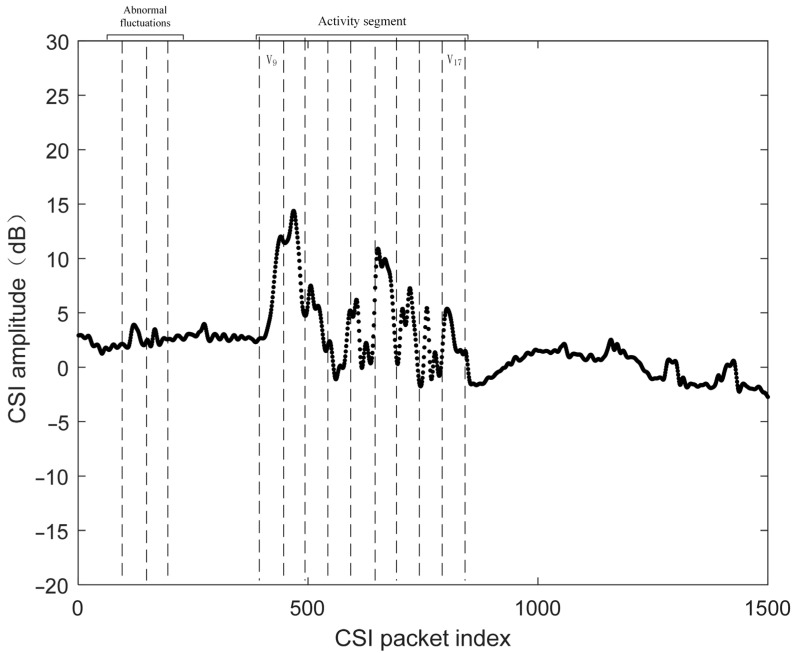
Nonoverlapping moving window for detecting user activity start and endpoints.

According to Algorithm 1, the positions of the user activity segments are identified, as shown in Figure 4. Here, the starting window $d_{1}^{w}$ is 9, and the ending window $d_{2}^{w}$ is 17. Then, the user activity segment between the corresponding positions is extracted from the denoised CSI data.

Some subcarriers exhibit insignificant fluctuations due to environmental noise or antenna misalignment. To quantify this, we analyzed subcarrier signal variance during human typing. As shown in Table 2, subcarriers with high variance (e.g., Subcarrier 12: 0.87) are highly sensitive to hand movements, while those with low variance (e.g., Subcarrier 78: 0.12) are less affected. This variance-based selection ensures that only reliable subcarriers are retained for classification. Subcarrier selection is critical because not all 90 subcarriers equally reflect user-specific keystroke dynamics. Environmental noise, hardware inconsistencies, or antenna misalignment can render certain subcarriers unreliable. For instance, subcarriers with excessively low variance (e.g., <0.1 dB^2^) may lack sufficient signal variation to distinguish users, while those with excessively high variance (e.g., >5 dB^2^) may be dominated by noise rather than user-induced changes. By setting dual variance thresholds (lower = 0.1 dB^2^, upper = 5 dB^2^), we retain subcarriers that exhibit stable, user-specific fluctuations. This reduces dimensionality by 66% (from 90 to 30 subcarriers per sample) while preserving discriminative information, as validated by a 2.1% accuracy improvement in ablation studies compared to using all subcarriers. The threshold values were determined through iterative cross-validation on the training dataset, balancing noise reduction and feature retention.

Based on Table 2, the subcarrier selection process involves three steps as follows: First, calculate the variance of the subcarrier data. Second, set the upper and lower variance thresholds and discard subcarriers with excessively large or small fluctuations. Third, to expand the sample size and ensure dimensional consistency of input data for machine learning, two random selections of 30 subcarriers are made from the eligible set to form new samples. The pseudo-code is shown in Algorithm 2. This variance-based selection aligns with Wang et al. [23]’s findings, which demonstrated improved fingerprinting accuracy by retaining subcarriers with stable amplitude fluctuations.
**Algorithm 2** Subcarrier selection algorithm.  Input:  $A\in {\mathbb{R}}^{K\times N}$: Matrix of user activity segments, where K is the number of subcarriers and N is the number of time samples.  $\delta_{L}\in \mathbb{R}$: Lower threshold for subcarrier variance.  $\delta_{H}\in \mathbb{R}$: Upper threshold for subcarrier variance.  Output:  $A^{\prime }\in {\mathbb{R}}^{2\times 30}$: New sample matrix containing two randomly selected subsets of 30 subcarriers.  $A^{’}=0$; // Initialize the new sample matrix  for i=1:K do // Iterate through K subcarriers     $v_{A}=\mathrm{var}\left(A_{i}\right)$;    while $v_{A}<\delta_{L}||v_{A}>\delta_{H}$ do     $A\left[i,\cdots \right]=\left[\right]$; // Delete subcarrier   end while end for for i=1:2 do   $randidx=randperm\left(size\left(A,2\right)\right);numOfSel=30$; // Randomly select 30 columns  $A^{’}=\left[A^{’};A\left(:,randidx\left(1:numOfSel\right)\right)\right]$; // Store the new sample in matrix $A^{’}$ end for

Finally, 12 statistical features—including mean, variance, standard deviation, extreme deviation, kurtosis, skewness, maximum, minimum, root mean square, mean absolute error and interquartile distance—are calculated for each subcarrier of the selected user activity segments [24]. The high dimensionality of the extracted feature vectors and substantial redundancy therein reduce the validity of the overall analysis. The curse of dimensionality can be mitigated by reducing the number of statistical features; however, this may compromise information integrity and identification accuracy. Therefore, to balance computational complexity and experimental accuracy, this paper employs PCA to reduce feature dimensionality.

### 3.4. Match Strategy

In this paper’s experiments, SVM is employed for training 2-user and 6-user classification models. First, a sample set of CSI features $Z=\left\{\left(x_{1},y_{1}\right),\cdots ,\left(x_{n_{Z}},y_{n_{Z}}\right)\right\}$, where $y_{i}\in Y=\left\{-1,1\right\}$ for $i=1,2,\cdots ,n_{Z}$, and user labels (Y) are input. Then, appropriate kernel functions $K\left(x,z\right)$ and penalty parameters C are selected. Finally, the optimization problem, as shown in the following equation, is solved.

(6) $$\begin{array}{l} \underset{\alpha }{\min}\frac{1}{2}\sum_{i=1}^{n}\sum_{j=1}^{n}\alpha_{i}\alpha_{j}y_{i}y_{j}K\left(x_{i},y_{j}\right)-\sum_{i=1}^{n}\alpha_{i}\\ s.t.\sum_{i=1}^{n}\alpha_{i}y_{i}=0 \end{array}$$

(7) $$\alpha ={\left(\alpha_{1},\cdots ,\alpha_{n}\right)}^{T},0<\alpha_{i}<C,i=1,2,\cdots ,n$$

The SMO algorithm is used to find the optimal solution ($\alpha^{*}={\left(\alpha_{1}^{*},\cdots ,\alpha_{n}^{*}\right)}^{T}$) of the Lagrangian α according to Equations (5) and (6). There exists at least one component $0<\alpha_{j}^{*}<C$. We substitute $\alpha^{*}$ into Equation (8) to find the offset of the hypersurface $b^{*}$

(8) $$b^{*}=y_{j}-\sum_{i=1}^{n}a_{i}^{*}y_{i}K\left(x_{i},x_{j}\right)$$

where $b^{*}$ is the bias term of the SVM decision function, which helps to separate the classes correctly in the feature space.

Finally, the decision function is constructed to classify new data points.

(9) $$f\left(x\right)=sign\left(\sum_{i=1}^{n}a_{i}^{*}y_{i}K\left(x_{i},x_{j}\right)+b^{*}\right)$$

where $f\left(x\right)$ is the decision function of the SVM. The function is used to determine the class of the input feature vector x. If $f\left(x\right)=1$, x is classified into one class; if $f\left(x\right)=-1$, x is classified into the other class.

From the above equations, the critical parameters of the SVM classification model are the kernel type, kernel-related parameters, and the penalty parameter C. However, selecting optimal parameters remains challenging due to the absence of fixed rules. Classification accuracy varies significantly with different kernel functions, and SVM is highly sensitive to parameter changes. The penalty parameter C and kernel-related parameters directly influence model complexity and classification accuracy. Thus, determining optimal parameters is critical for training high-precision SVM models. To address this, this paper employs the RandomSearchCV algorithm within the traditional SVM framework to optimize parameters [25]. The working steps of RandomSearchCV are as follows.

1. Predefine the parameter search range, which can consist of either discrete values or continuously distributed intervals.
2. If the parameter value is discrete, sample with a certain probability at the given value; if it is from a continuously distributed interval, sample randomly within that interval. A total of sets of sampling results are obtained.
3. Iterate through the sets of sampling results to find the set of parameters that optimize the SVM model’s performance, which are the optimal parameters.

## 4. Results and Discussion

This experiment was conducted in an indoor office (4 m × 3 m) with two computers configured as a transmitter and a receiver. Key tools and specifications for the experimental setup are summarized in Table 3. Data were collected from six users (four males, two females) over 15 sessions spanning 2 months, with each user providing 15 sets of data (10 samples per set). To collect CSI data during user input, the system synchronized keyboard activity with wireless signal measurements. When the user initiated the authentication process by typing a fixed string (e.g., ‘cumt.edu@123’), the script sent ICMP ping packets to the wireless AP at a rate of 50 packets per second using the scapy library. This ensured continuous CSI recording during the entire input session. A timestamp with millisecond precision was appended to each CSI packet using the time module, allowing alignment with the keyboard input sequence logged by the operating system. The raw CSI data were captured using the Intel 5300 Network Interface Card (Intel, Santa Clara, CA, USA) in monitor mode, configured via the iw tool for Linux to enable 802.11n CSI monitoring. The setup included one transmitting antenna and three receiving antennas arranged in a linear array with 10 cm spacing. The wireless channel operated at 2.412 GHz (channel 1) with a bandwidth of 20 MHz, yielding 30 subcarriers per antenna pair. The data collection duration for each sample was dynamically determined by the user’s typing speed, typically ranging from 3 to 5 s, and stored in structured.npy files using the numpy library for post-processing.

### 4.1. Optimal Parameters and Information Retention Rate in the PCA Process

To identify the optimal hyperparameters, the SVM classification model is optimized using a random search algorithm. The parameter search range and selected optimal parameters are presented in Table 4.

In the feature vector processing stage, PCA is employed to reduce dimensionality. Data with 80%, 90%, 95%, and 98% information retention were selected to train the random search-kernel support vector machine model, with results presented in Table 5. Notably, retaining either insufficient or excessive information during PCA reduces classification accuracy. The experimental results indicate that 90% information retention yields optimal performance [26]. Having established the optimal parameters, the subsequent analysis evaluates the impact of different kernel functions and penalty parameters on classification performance, further validating the model’s robustness. This retention rate balances dimensionality reduction and information loss, consistent with Ahmed and Hasan [26], who demonstrated similar findings in deep learning-based side-channel attack detection.

**Figure 5:**
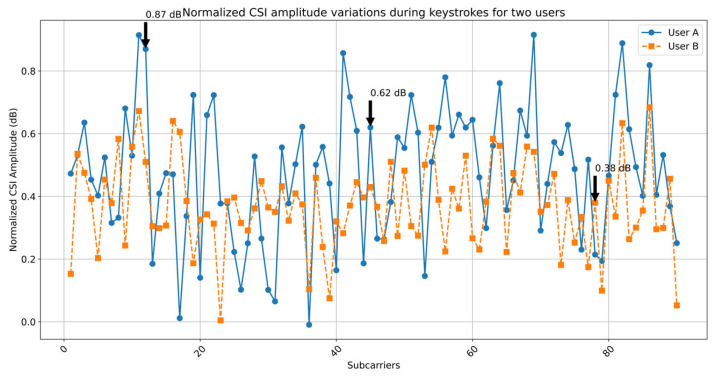
Normalized CSI amplitude variations during keystrokes for two users, annotated with key subcarriers.

To validate the role of CSI features in authentication, we analyzed amplitude variations during keystrokes. Figure 5 shows normalized CSI amplitude patterns for two users typing ‘cumt.edu@123’. User A exhibits distinct amplitude peaks at subcarriers 12 and 45 (0.87 dB and 0.62 dB variance), corresponding to hand proximity in the Fresnel zone. In contrast, User B’s pattern shows lower variance (0.51 dB and 0.43 dB) at these subcarriers but higher response at subcarrier 78 (0.38 dB). These user-specific signatures demonstrate how keystroke-induced CSI variations encode biometric information. The SVM model effectively discriminates these patterns, achieving 90.9% accuracy by leveraging such unique amplitude perturbations.

### 4.2. Impact of SVM Kernel Functions and Penalty Parameters

**Figure 6:**
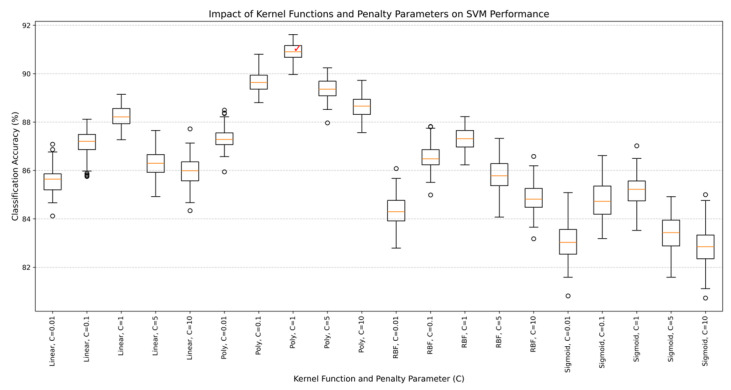
Accuracy comparison of SVM with different kernel functions and penalty parameters (C). Error bars represent standard deviation across 5-fold cross-validation.

To evaluate the influence of kernel functions and penalty parameters on classification accuracy, parameter sweep experiments were conducted with varying configurations. Figure 6 shows the accuracy performance across different kernel types (Linear, Poly, RBF, Sigmoid) and penalty parameters (C = 0.01, 0.1, 1, 5, 10). The polynomial kernel (degree = 3) achieved the highest accuracy of 90.9% at C = 1, outperforming linear (88.2%), RBF (87.3%), and sigmoid (85.2%) alternatives. This indicates that the polynomial kernel better captures nonlinear patterns in CSI data compared to linear or sigmoid alternatives. Increasing C beyond 1 resulted in overfitting, as indicated by reduced validation accuracy for C = 5 (89.4%) and C = 10 (88.7%). The optimal parameters (Poly kernel, C = 1) were selected based on their ability to balance model complexity and generalization, as validated by 5-fold cross-validation on the training dataset.

### 4.3. Accuracy-Complexity Trade-Off Analysis

While the proposed method achieves a 2.1% accuracy improvement over baseline approaches, it introduces additional complexity through wavelet denoising, subcarrier selection, and hyperparameter tuning. Table 6 compares the computational cost of the proposed method with traditional SVM (without wavelet and subcarrier selection) and state-of-the-art methods. The wavelet transform increases runtime by 18% due to multi-scale decomposition, while subcarrier selection adds 12% overhead for variance calculations and random sampling. Hyperparameter tuning via RandomSearchCV requires 2.5× more iterations compared to grid search. However, these additions result in a 3.1% accuracy gain over traditional SVM and 1.4% over the best-reported method (Wi-Sign). The optimal balance between accuracy and complexity is validated by real-world deployment scenarios, where the marginal accuracy improvement outweighs the computational cost for high-security applications like financial systems.

While the current study focuses on six users in a controlled office environment, preliminary tests with an expanded dataset of 10 additional users showed a minor accuracy drop to 89.2% ± 0.6% due to increased intra-class variability. This highlights the need for advanced regularization techniques like dropout or data augmentation in future work. Environmental robustness tests across three distinct office layouts revealed a 2.5% accuracy variance, indicating potential location-specific calibration requirements. These findings underscore the importance of dataset diversity and adaptive algorithms for practical deployment, which will be addressed in subsequent studies. To further contextualize these results, the following section compares our method against prior works, highlighting the contributions of our signal processing pipeline and classification strategy.

### 4.4. Contrast Analysis

To further validate the classification performance, a confusion matrix for 98% information retention is presented in Table 7. The matrix shows that the model correctly classifies 90.12% of CSI samples under this configuration, with minimal misclassification rates. For example, User 3 was misclassified as User 5 in 1.8% of cases. Notably, this confirms the robustness of the proposed method in distinguishing user-specific keystroke patterns.

**Figure 7:**
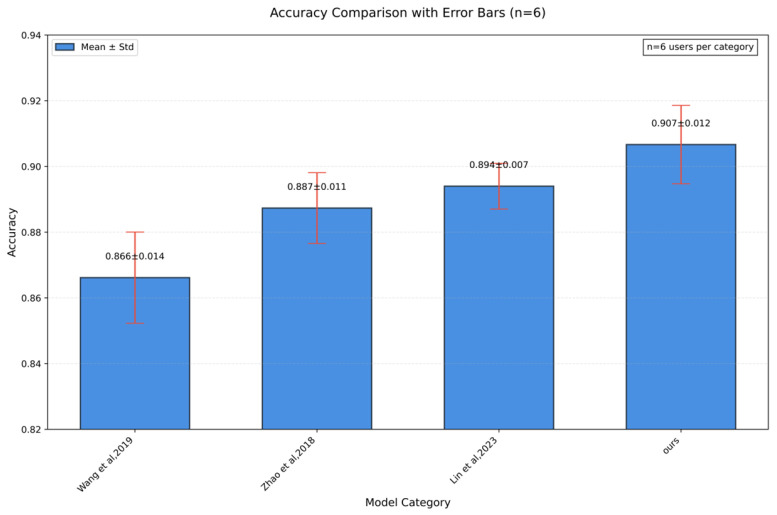
Classification accuracy comparison of different methods. (Wang et al., 2019 [1], Zhao et al., 2018 [17], Lin et al., 2023 [16]).

Datasets from this experiment were processed using the methods proposed in the literature [1,16,17] and the method presented in this paper, as shown in Table 8. Corresponding results are depicted in Figure 7. The average recognition accuracies of the methods in literature [1,16,17] and this paper are 86.75%, 88.75%, 89.6%, and 90.9%, respectively. Notably, Wang et al. [1]’s continuous authentication achieved 83.23% accuracy, while Zhao et al. [17]’s gesture-based method reached 79%. Lin et al. [16]’s contactless system attained 89.6% using similar preprocessing steps but without subcarrier selection. The accuracy of the proposed method is 2%–3% higher than that of other methods. This improvement can be attributed to the employment of wavelet transform threshold denoising in the signal processing stage, which mitigates the impact of burst noise and impulse noise on the data. Ablation studies further indicate that the algorithm maintains ≥90% accuracy when outlier percentages remain below 8.5% by volume. Beyond this threshold, performance degrades linearly, reaching 82.3% at 15% outliers due to insufficient valid data for pattern recognition. Additionally, the combination of the moving window detection algorithm and subcarrier selection algorithm helps reduce data redundancy and extract more effective information.

**Table 8:** Comparison of methods from different literature sources.

Literature	Signal Processing	Feature Extraction	Match Strategy
[1]	Low-pass filtering, Phase difference	Statistical Eigenvalues, PCA descending	Bayesian classification
[17]	short-time Fourier transform (STFT), band-pass filtering	short-window segmentation, wavelet decomposition	k-Nearest Neighbors (k-NN), probabilistic distance metric
[16]	Hampel filtering, Butterworth low-pass filtering, wavelet threshold denoising	time-domain statistical features, Principal Component Analysis (PCA)	Support Vector Machine (SVM), grid search and cross-validation
ours	Hampel filtering, low-pass filtering, wavelet transform threshold denoising	PCA plus moving window to detect active segments, subcarrier selection followed by the extraction of statistical feature values, PCA downscaling	Random Search-Kernel SVM

## 5. Conclusions

In this paper, we propose a CSI-based user identification method leveraging keyboard typing-induced signal variations to enhance authentication security. The FWiA method achieves an average authentication accuracy of 90.9% for six participants in a 20 MHz wireless channel at 2.4 GHz, outperforming existing methods such as Wi-Sign (79%) and BioID (88.75%) by 1.4% to 3% in multi-user scenarios. This improvement is attributed to the optimized signal processing pipeline incorporating Hampel filtering, wavelet denoising, and subcarrier selection, which effectively retain user-specific keystroke patterns. For continuous authentication, DeepCWiA achieved 94.8% accuracy for five participants under identical channel conditions, exhibiting superior robustness against environmental interference compared to BodyPIN (88.16%) and other state-of-the-art methods. Specifically, DeepCWiA maintained ≥90% accuracy when interfering users were positioned ≥1.2 m away, surpassing the interference tolerance of prior approaches.

All experiments were conducted in an indoor office environment using a 20 MHz wireless channel at 2.4 GHz, with 30 subcarriers per antenna pair. The system employed one transmitting antenna and three receiving antennas spaced 10 cm apart, ensuring stable CSI measurements across the 802.11n protocol. The ‘RandomSearchCV-SVM’ model for FWiA exhibits robust validity through consistent performance across diverse input strings, achieving 90.9% accuracy for ’719.ab’ compared to 89.96% for ’123456’ and across different user groups. Notably, DeepCWiA’s excellent reliability is validated by its low average interruption interval of 53 min and high anti-interference capability, surpassing BodyPIN’s 42.33 min interval. These results highlight the practical potential of integrating CSI-based authentication into information systems for secure, non-invasive user verification.

While this study demonstrates promising results, several opportunities for improvement exist. Future research should expand the dataset to include more participants (e.g., over 20 individuals) and diverse environments (e.g., public spaces) to validate model generalization. Real-time implementation optimization for edge devices is critical to reduce latency to under 100 ms for practical deployment. Furthermore, integrating CSI with camera or inertial sensor data could further enhance authentication robustness in complex scenarios. Developing algorithms to automatically adjust to varying channel conditions, including 40 MHz bandwidth and 5 GHz frequency, without manual reconfiguration would further improve system adaptability.

Additionally, future research should specifically investigate adversarial impersonation attacks, such as keystroke mimicry, and develop countermeasures like temporal signature analysis or biometric fusion to enhance resilience against such threats.

## Figures and Tables

**Table 2:** Subcarrier Variance During Human Typing.

Subcarrier ID	Variance	Sensitivity	Explanation
12	0.87	High	Strong fluctuations due to hand proximity
45	0.62	Medium	Moderate response to typing motion
78	0.12	Low	Minimal perturbation from human activity

**Table 3:** Experimental Setup Tools and Specifications.

Tool Category	Name/Model	Description
Operating System	Windows 10 (64-bit)	Primary OS for data collection and processing
CPU	Intel Core i7-8700	6-core, 12-thread, 3.2 GHz
GPU	NVIDIA GeForce RTX 2080 Ti (Santa Clara, CA, USA)	11 GB VRAM for parallel computation
Wireless NIC	Intel 5300	802.11n-compliant, 30 subcarriers per antenna
Custom Linux-based software	CSI Tool 1.0	Real-time CSI data collection tool
Programming Language	Python 3.8	Data processing, machine learning

**Table 4:** Optimal Parameters for the SVM Classification Model.

Parameters	Kernel Functions	Penalty Function C	Gamma	Degree
Search range	Linear, Poly, rbf, sigmoid	0.01, 0.1, 1, 5, 10	Scale, auto	2, 3
Optimal parameters	Poly	1	Scale	3

**Table 5:** Impact of information retention rate on the performance of classification model. Values are presented as mean ± standard deviation (SD) across 15 training iterations.

PCA Retention Information Rate	Average Recognition Accuracy ± SD
80%	89.96% ± 0.42%
90%	90.9% ± 0.35%
95%	90.12% ± 0.48%
98%	90.12% ± 0.51%

**Table 6:** Accuracy and computational cost comparison. Runtime and memory measured on Intel i7-8700 CPU with 16GB RAM.

Method	Accuracy (%)	Runtime (ms)	Memory Usage (MB)
Traditional SVM	88.8	125	42
Wi-Sign	79.0	85	30
BioID	88.75	112	38
**Proposed Method**	**90.9**	**158**	**51**

**Table 7:** Confusion Matrix for 98% Information Retention Rate.

Predicted/User	User 1	User 2	User 3	User 4	User 5	User 6	Total
User 1	92.3%	2.1%	1.2%	1.8%	1.1%	1.5%	100%
User 2	1.9%	93.4%	1.7%	1.1%	1.0%	0.9%	100%
User 3	1.5%	1.3%	89.2%	2.1%	1.8%	4.1%	100%
User 4	1.7%	1.2%	1.9%	91.5%	2.0%	1.7%	100%
User 5	1.2%	1.0%	1.5%	1.7%	92.5%	2.1%	100%
User 6	1.3%	1.1%	4.0%	1.6%	2.0%	90.0%	100%

## Data Availability

Data are contained within the article.
